# Eugenol and *Aloe vera* blended natural wax-based coating for preserving postharvest quality of Kaji lemon (*Citrus jambhiri*)

**DOI:** 10.1016/j.fochx.2024.101349

**Published:** 2024-04-06

**Authors:** Bhaswati Das, L. Susmita Devi, Joydeep Dutta, Santosh Kumar

**Affiliations:** a Department of Food Engineering and Technology, Central Institute of Technology Kokrajhar, Kokrajhar, Assam 783370, India; b Functional NanoMaterials Group, Department of Applied Physics, School of Engineering Sciences, KTH Royal Institute of Technology, Hannes Alfvéns väg 12, 114 19 Stockholm, Sweden

**Keywords:** Carnauba wax, Citrus fruit, Edible coating, Essential oil, Innovative preservation technique, Shelf-life, Shellac wax

## Abstract

Edible coatings on fruits and vegetables preserve postharvest quality by reducing water loss and lowering respiration, and metabolic activities. The primary objectives of this study were to develop composite coating formulations using natural waxes (carnauba and shellac wax), eugenol nanoemulsion, and *Aloe vera* gel, and assess the potential impacts of the coating formulations on the postharvest quality and shelf-life of the Kaji lemon. The results show that eugenol nanoemulsion and *Aloe vera* gel enhanced the physico-chemical, antimicrobial and antioxidant properties of the developed coating. Notably, the fruits coated with optimized nanocomposite of wax with eugenol and *aloe vera* gel inclusion (SW + CW/EuNE-20/AVG-2) showed the lowest weight loss (16.56%), while the coatings of wax with only *aloe vera* gel (SW + CW/AVG-2) exhibited the highest firmness (48 N), in contrast to the control fruit, which had 27.33% weight loss and 9.6 N firmness after 28 days of storage, respectively.

## Highlights

• Natural wax-based coatings containing eugenol and *Aloe vera* gel were prepared.
• The prepared coating formulations showed good antimicrobial activities.
• Coating on Kaji lemon improved its quality and extended shelf-life up to 20 days.
• Incorporation of eugenol nanoemulsion and *Aloe vera* improved coating functionalities.

## 1 Introduction

In tropical regions, postharvest losses in citrus fruits pose a significant challenge due to their rapid deterioration during handling, storage, and transport (Strano et al., 2022). Lemons, being non-climacteric fruits, emit low levels of CO_2_ and ethylene during postharvest storage that led to moderate softening or compositional alterations during storage and transport. However, the postharvest life of citrus fruits can be prolonged by addressing challenges of weight losses, primarily caused by transpiration and respiration, as well as the reduction of color changes and fungal growth (Nasrin et al., 2023). To achieve such goals, various postharvest treatments can be employed, including the use of growth regulators, waxing, low-temperature storage, fungicides, chemicals, oil coatings, irradiation, and different packaging materials. Edible coatings are considered a safe option for preservation of fruits, forming a thin layer that limits gas exchange and water loss, thereby extending storage life by increasing CO_2_ and reducing O_2_ levels (Basumatary, Kalita, Katiyar, Mukherjee, & Kumar, 2022). While conventional synthetic wax coatings derived from petroleum resources can extend postharvest life, their prolonged use has adverse impacts on both the environment and human health (Bhandari, Bika, Subedi, & Pandey, 2022). Consequently, there is a growing interest in developing novel preservation technologies that are environmentally friendly and utilize readily available, cost-effective alternatives. Lipid-based edible coatings derived from natural materials offer a promising solution, forming a barrier against water vapor and enhancing fruit appearance and shelf-life for maintaining quality during storage (Miranda et al., 2022).

Lipid-based biopolymers are used directly or incorporated with other biopolymers or active agents as composite coatings and films (Mohamed, El-Sakhawy, & El-Sakhawy, 2020). Among the lipids, carnauba wax (CW) and shellac wax (SW) have been extensively studied due to their inherent antioxidant activity, abundant availability, and low cost. (Devi, Mukherjee, Dutta, & Kumar, 2023). Moreover, as natural polymers, they exhibit excellent biocompatibility and biodegradability, making them environmentally friendly alternatives to synthetic waxes. Both CW and SW are considered safe by the US Food and Drug Administration for use in edible coatings and as food additives, further enhancing their appeal for food packaging and preservation applications (Devi et al., 2023). CW, derived from Brazilian palm tree (*Copernicia prunifera*) leaves, is a hydrophobic wax known for its exceptional water barrier properties and possesses natural antifungal and antibacterial properties (de Freitas et al., 2019). It is primarily composed of aromatic acids, esters, triterpene diols, free alcohols, hydrocarbons, aliphatic acids, and free ω-hydrocarboxylic acids, with a low solubility and high melting point (82–86 °C). Shellac, known as Kerria lacca, is a natural mixture of resins produced by lac insects (K. Li et al., 2022). Shellac is a natural hydrophobic polymer, containing hydroxy fatty acids and sesquiterpene acids (Yuan et al., 2021). Historically, SW was used as a protective finish by painters and as natural dye for leather and silk. Gradually shellac has found applications in the food and pharmaceutical sectors due to its enteric properties and excellent characteristics such as solubility in both spirit and aqueous alkaline solvents, better film-forming ability, adhesion, bonding, thermo-plasticity, and resistance to water (Yuan et al., 2021).

The utilization of plant extracts as active coating additives offers significant potential for preserving the physical, oxidative, and microbiological quality of fruits. Eugenol (4-allyl-2-methoxyphenol), a key component of clove oil and other essential oils, exhibits good anti-inflammatory, antioxidant, and antimicrobial properties (Liu et al., 2021). However, its low solubility in water and high volatility limits its practical use. Enhancing the stability and antibacterial effectiveness of eugenol can be achieved by creating highly dispersed and permeable nanoemulsions (Fu et al., 2022). Nanoemulsions, colloidal dispersions with excellent kinetic stability, have droplet diameters ranging from 30 nm to 200 nm, mitigating aggregation and flocculation, thus improving emulsion stability (Salvia-Trujillo, Soliva-Fortuny, Rojas-Graü, McClements, & Martín-Belloso, 2017). Meanwhile, *Aloe vera* gel (AVG), known for its natural antimicrobial properties and antioxidant effects, has been used for centuries (Kumar et al., 2022). Incorporating AVG into edible coatings enhances the visual appearance of food, reduces moisture loss, maintains firmness, lowers respiration rate, delays browning, and inhibits microbial growth (Ul Hasan et al., 2021). Several studies have investigated the application of shellac and carnauba wax, either alone or combined with essential oils, for preserving fruits such as papaya (J. G. Oliveira Filho et al., 2023), litchi (Nanglia et al., 2022), and mango (Ma et al., 2021). However, to the best of our knowledge, there is limited research on the development of composite coating formulations of shellac and carnauba wax incorporated with eugenol nanoemulsion and *Aloe vera* gel, and its application for preserving and prolonging the shelf life of citrus fruits. Despite its high potential as a safe and more natural alternative to conventional synthetic was-based coating, this area remains underexplored. Therefore, this study mainly focusses on development, characterisation and application of shellac wax, carnauba wax, eugenol nanoemusion, and *Aloe vera* gel-based active coating formulations for preservation of Kaji lemon. The effects of the coatings on the postharvest shelf-life and quality including weight loss, firmness, total soluble solids, titratable acidity, ascorbic acid content, and physical attributes of Kaji lemon fruit were evaluated.

## 2 Material and methods

### 2.1 Collection of materials

Shellac flake was obtained from HiMedia Pvt. Ltd. Mumbai, India. Carnauba wax No-1 yellow, Tween 80 and Eugenol were procured from Sigma-Aldrich. Mueller Hinton Agar media, Iodine, Potassium iodide was purchased from HiMedia (Mumbai, India) and sodium hypochlorite (NaOCl) solution was procured from Avantor Performance Materials, Maharashtra, India. Fresh lemon of similar color, size, and maturity, free of any visual damage were procured from a local farmer of Kokrajhar district, Assam, India. Aloe gel (AG) was extracted by scraping the outer epidermis of fresh Aloe leaves, which was obtained locally. The bacterial strains namely *Staphylococcus aureus*, *Escherichia coli*, *Bacillus subtilis*, and *Alcaligenes faecalis* were used for antibacterial study. The fungal strains *Saccharomyces cerevisiae* and *Rhizopus stolonifer* were obtained from the microbial type culture collection (MTCC) and gene bank, CSIR-Institute of Microbial Technology and the fungi were activated on the potato dextrose agar (PDA).

### 2.2 Preparation of eugenol nanoemulsion (EuNE)

The eugenol nanoemulsion was prepared using the method described by Basumatary et al., 2022 with slight modification (Basumatary, Mukherjee, Katiyar, Dutta, & Kumar, 2022). Tween 80 was used as a non-ionic surfactant and emulsifier for the preparation of a eugenol oil/water nanoemulsion. Eugenol is sensitive to humidity, light, and temperature, so the primary emulsion of eugenol was prepared at ambient temperature, followed by ultra-sonication to form eugenol nano-emulsion (EuNE). Different concentrations of Tween 80 i.e., 5, 10, 15, 20 and 30%, *v*/v) were used as emulsifying agent. Solutions of tween 80 were prepared in double distilled water (ddH_2_O) under continuous magnetic stirring (REMI, India) for 10 min at 1200 rpm. Then, dropwise 10% (*v*/v) eugenol was added into the prepared solution while mixing constantly for another 10 min at 1200 rpm. Primary emulsion of eugenol was then nano-emulsified using ultra-sonication at 20 KHz and 750 W power output for 5 min at temperature below 25 °C using a probe sonicator (Vibra cell VCX-750, Sonics & Materials, Inc., Newton, USA).

### 2.3 Characterisation of the synthesized EuNE

#### 2.3.1 Determination of pH and viscosity

The pH of the nanoemulsions was evaluated using a pH meter (ION 2700, Thermo Fisher Scientific India Pvt. Ltd., Mumbai, India) at 25 °C in ambient condition. The EuNE viscosity of the prepared nanoemulsions was determined by using viscometer (DV3T, Brookfield Ametek, USA) at ambient condition with cylindrical spindle (LV5) at a speed of 80 rpm (Devi et al., 2023).

#### 2.3.2 Thermodynamic stability study

The thermodynamic stability of the prepared nanoemulsions under alternating heating and cooling conditions were assessed using the method described by Sharma, Kaur, & Khatkar, 2021 with slight modification (Sharma et al., 2021). The formulated emulsion was centrifuged for 30 min at 3500 rpm to evaluate any phase separation and stability. The emulsions were then treated through heating-cooling cycle at 40 °C and 4 °C, alternately for 48 h. Then, the freeze thaw cycle was carried out by holding the emulsion at −21 °C and 25 °C for 48 h at each temperature to confirm the stability of emulsions with fluctuating temperature. The formulations that passed the thermodynamic stress tests were used to conduct further optimization of the composites.

#### 2.3.3 Determination of antioxidant activity

The methodology of Adebiyi et al., 2017 was employed with minor changes using 2, 2-diphenyl-1-picrylhydrazyl (DPPH) for determining the antioxidant capacity of the prepared nanoemulsions (Sharma et al., 2021). Briefly, 100 mL DPPH solution was prepared, and 3.9 mL of it was added into 100 mL methanol, which was added to 0.1 mL of the sample. After that, the solution was incubated in the dark for 45 min at room temperature followed by measurement of optical absorbance at 517 nm with a spectrophotometer (2375 Double Beam, Electronic India, Haryana, India). The ability of DPPH radical to scavenge is represented as inhibition % as shown in equation-1.

(1) $$\text{DPPH scavenging activity}\;\left(\%\right)=Ab-As/Ab\times 100$$

where, *A*_b_ and *A*_s_ is the absorbance of the blank and the sample, respectively.

#### 2.3.4 Evaluation of antibacterial and antifungal properties

The antibacterial and antifungal activities of the prepared EuNEs was assessed using the modified agar well diffusion method (Basumatary, Mukherjee, et al., 2022). For antibacterial analysis, the bacterial strains namely *Staphylococcus aureus*, *Escherichia coli*, and *Bacillus subtilis* were grown in nutrient agar media at 37 °C for 24 h before the experiments. Then, the bacterial strains were inoculated in a sterilised 10 mL 0.85% sodium chloride (NaCl) solution for preparing 0.5 McFarland standards. Then, 200 μL of each test bacterial suspension was spread over earlier solidified and sterilised Mueller Hinton agar media in a biosafety cabinet (Airstream, Esco Lifescience Group, Singapore). Wells were created in the middle of each Petri-dish and filled up with 200 μL EuNE and then incubated at 37 °C for 24 h. The zone of inhibition (ZoI) was determined using electronic digital calliper (Hitech Survey Tools Pvt. Ltd., Haryana, India). For antifungal examination of EuNEs against *Saccharomyces cerevisiae and Rhizopus stolonifer*, similar protocol as antibacterial test was used except using PDA instead of Mueller Hinton agar as the growth media.

### 2.4 Preparation of coating formulations

Shellac and carnauba wax-based edible coating formulations combined with EuNE and AVG were developed by adopting the technique described by Singh et al., 2016 with minor changes (Singh et al., 2016). Initially, the shellac, and carnauba wax (1:1) i.e., 0.5%, *w*/*v* of each were dissolved in alkaline solvent (0.5% ammonium hydroxide) at 95 °C through continuous stirring at 3000 rpm on a magnetic stirrer (REMI, Mumbai, India) by using oleic acid as an emulsifier. Thereafter, 10%, *v*/v of EuNE-20 and 2%, *w*/*v* of AVG were added into it. Thus, four different coating formulations namely SW + CW, SW + CW/EuNE-20, SW + CW/AVG-2, and SW + CW/EuNE-20/AVG-2 were prepared as summarized in Table 1. In addition, only 2%, w/v of AVG solution was also prepared separately, and used as another coating formulation.

**Table 1:** Preparation of carnauba wax and shellac wax-based coating formulations.

Coating formulation	SW %, w/v	CW %, w/v	Eugenol nanoemulsion (EuNE-20) %, v/v	*Aloe vera* gel (AVG) %, w/v
SW + CW	0.5	0.5	_	_
SW + CW/EuNE-20	0.5	0.5	10	_
SW + CW/AVG-2	0.5	0.5	_	2
SW + CW/EuNE-20/AVG-2	0.5	0.5	10	2
AVG-2	_	_	_	2

[SW; Shellac wax, CW; Carnauba wax, EuNE-20; Eugenol nanoemulsion with 20% (*v*/v) Tween 80, and AVG; *Aloe vera* gel].

### 2.5 Application of coating on fresh lemon

The developed formulations were applied on the fresh lemon following the procedure described earlier (Devi et al., 2023). Eighty-five (85) whole, fresh lemon locally called as Kaji Nemu (*Citrus jambhiri*) of similar size and maturity were used in this study to assess the effects of coating on the shelf-life and quality of the fruit. They were sanitized, washed, and likewise separated into 6 groups. Five lemon groups were then coated with the 5-different coating formulations by dip-coating, while the one group of lemon without coating was taken as a control sample group. The coated and uncoated lemon samples were dried in air at room temperature for 3 h before being stored in the ambient for 28 days for the study. Weight loss, total soluble solids (TSS), pH, firmness, antioxidant activity, titratable acidity (TA), ascorbic acids (Vitamin C), and physical attributes of treated lemons were evaluated at every 4-days interval.

### 2.6 Evaluation of effects of coating on quality of the coated lemon

#### 2.6.1 Weight loss and firmness

Physiological reductions in weight were calculated by weighing the lemon before and after the specific time of storage similar to preliminary experiments reported by Zhang, Chen, Lai, Wang, & Yang, 2018 (Zhang et al., 2018). The percentage reduction in weight of the fruit samples was measured gravimetrically following Eq. 2, where, W_f_ and W_i_ are the lemon's final and initial weights, respectively.

(2) $$\text{Weight loss}=\left(W_{i}–W_{f}\right)/W_{i}\times 100$$

The level of firmness of the lemon samples were determined by texture analyser (TA. XT Plus, Stable MicroSystem Ltd., UK) with cylindrical probe of 6 mm diameter at an operating velocity of 10 mm/s^−1^. Each sample was examined three times at three distinct points (below, upper and either right or left), and the highest force in Newton (N) was measured from the force versus time curve.

#### 2.6.2 pH, TA, TSS, and TSS/TA

Lemon samples were homogenized using a manual juicer, filtered using cheesecloth, and were immediately used for measuring pH, TA and TSS. pH of the juice was assessed using pH-meter. The total acidity was measured by titration method as described by Panahirad, Naghshiband-Hassani, Bergin, Katam, & Mahna, 2020, where lemon juice was quantified through titration with 0.1 N NaOH up to pH 8.1 and expressed as grams of citric acid equivalents per liter of juice (Panahirad et al., 2020). Using a digital refractometer (Atago, Tokyo, Japan) TSS content was determined and expressed as a percentage of soluble solids. The maturity index was defined following Basumatary, Mukherjee, Katiyar, Kumar, & Dutta, 2021 as the ratio of TSS/TA (Basumatary et al., 2021).

#### 2.6.3 Vitamin C content

The ascorbic acid content in the juice samples was evaluated using iodine titration method as described by Alkanan, Altemimi, Al-Hilphy, Cacciola, & Ibrahim, 2021 (Alkanan et al., 2021). Briefly, 25 mL of juice samples (of control or treated lemon) instantly after homogenization was mixed with 35 mL of starch-sulphuric acid solution and it was titrated against previously standardized 0.1 M iodine solution. Endpoint of the titration was recognized as the initial permanent color change recorded to dark blue-black color caused due to starch‑iodine complexation. The ascorbic acid content in juice samples was determined using equation-3.

(3) $$\text{Ascorbic acid}\;\left(\frac{\mathrm{mg}}{100}\mathrm{mL}\right)=\frac{\mathrm{Net}\;\mathrm{mL}\;\text{titrant}}{\mathrm{mL}\;\text{of sample}}\times 880.6$$

where, net mL titrant = mL titrant of the sample - mL titrant of the blank.

#### 2.6.4 Sensory analysis

Sensory analyses of the treated lemon were conducted by adopting the protocol of Saleem et al., 2021 with slight variations (Saleem et al., 2021). Untrained sensory panel i.e., students and faculty of Central Institute of Technology Kokrajhar of age ranging between 18 and 45 years were recruited and the panel were trained for identifying the desired characteristics, for example the visual appearance, aroma, taste, and overall acceptability prior to conducting the experiment. Sensory evaluation was conducted at ambient conditions (25 °C ± 2.0), and humidity (60% ± 5.0) inside the laboratory of dimension of 15 m × 20 m in natural light. For evaluating relative approval scores, a 9-point hedonic scale was used in which score of 9 for like extremely, 5 for neither like nor dislike and 1 for dislike extremely were considered.

### 2.7 Statistical analysis

The different experimental data were displayed as the mean value SD and subjected to ANOVA with duration of storage and different coating formulation as sources of variation. Mean among the various treatments were achieved by Tukey's test at a significance level of *p* < 0.05. OriginPro 9.0 software was used for analysing and it was carried out in triplicates.

## 3 Results and discussion

### 3.1 Characterisations of eugenol nanoemulsion (EuNE)

The effect of Tween 80 amount (i.e., 5, 10, 15, 20 and 30%, *v*/v) on viscosity, pH, antioxidant, and stability of the nano-emulsions are summarized in Table 2. The viscosity of the nanoemulsion increased with higher Tween 80 concentrations. Nanoemulsions with the minimum Tween 80 concentration (5%) had the lowest viscosity (5 cP), while those with the maximum Tween 80 concentration (30%) possessed the highest viscosity (145 cP). This is due to the rise in surfactant concentration leading to the formation of a dense, charged interfacial layer that reduce droplet aggregation, thereby ensuring high viscosity (Kaur, Singh, & Sharma, 2021). These findings are in agreement with a previous study, where the viscosity of the nanoemulsion was reported to increase from 1.12 to 1.57 mPa.s when concentration of surfactant (Tween 80) was raised from 2 to 8% (Gasa-Falcon, Odriozola-Serrano, Oms-Oliu, & Martín-Belloso, 2019). Similarly, a cinnamon-based nanoemulsion with the oil to surfactant ratio (1:2) exhibited the highest viscosity of 4 cP, confirming that an increase in surfactant concentration increases the viscosity of nanoemulsions (Kaur et al., 2021). pH of the nanoemulsions decreased slightly with increasing Tween 80 concentration in the admixture, ranging from 5.6 for EuNE-5 to 4.9 for both EuNE-20 and EuNE-30, with no significant differences observed. The developed nanoemulsions remained stable during storage at room temperature. Several earlier studies have demonstrated stable pH levels using Tween 80 as a surfactant in nanoemulsions across a wide range of temperatures, from 30 to 90 °C (Kaur et al., 2021; Mehmood & Ahmed, 2020). These findings also suggest that Tween 80, as a surfactant for nanoemulsion preparation, serves as an effective delivery system for incorporating essential oils into coating formulations. The DPPH inhibition % of the nanoemulsions samples increased with the increase in Tween 80 concentration, could be due to the improved solubility and increased surface area of the suspended droplets (da Silva, do Rosário, Neto, Lelis, & Conte-Junior, 2023). However, the EuNE-20 coating formulation exhibited the highest antioxidant capacity for scavenging DPPH radicals with 27.06% DPPH inhibition rate and EuNE-5 showed the lowest antioxidant capacity, with only 20.76% DPPH inhibition rate. In studies by Basumatary & Kumar (2024), eugenol nanoemulsions were found to exhibit good antioxidant activities, with no significant differences noted among the nanoemulsions prepared with different Tween 80 concentrations (Basumatary & Kumar, 2024). The antioxidant activities are essential for the coating formulation that help in preserving the coated food and extending their shelf-life. The thermodynamic stability study of the nanoemulsion samples on different stress conditions, such as heating, cooling, centrifugation, and freeze-thaw cycles were performed, and the samples EuNE-10, EuNE-15, and EuNE-20 were found to be stable showing no indications of phase separation during stress tests and room temperature storage, while EuNE-5, and EuNE-30 were found to be noticeably unstable.

**Table 2:** Characterisations of the prepared EuNEs.

Nanoemulsion	Viscosity (cP)	pH	DPPH inhibition (%)	Stability
EuNE-5	5.0 ± 0.57^a^	5.61 ± 0.02^a^	20.76 ± 0.25^a^	Unstable
EuNE-10	7.5 ± 0.31^b^	5.10 ± 0.01^b^	22.41 ± 0.57^b^	Stable
EuNE-15	10.1 ± 0.32^c^	5.23 ± 0.08^c^	22.86 ± 0.43^b^	Stable
EuNE-20	60.1 ± 0.51^d^	4.93 ± 0.01^d^	27.06 ± 0.38^c^	Stable
EuNE-30	105.0 ± 0.15^e^	4.97 ± 0.06^d^	24.19 ± 0.28^d^	Unstable

The values are of triplicate assessment given as means ± SD, and the lower-case superscript letters indicate that they are significantly different (*p* < 0.05). [EuNE; Eugenol nanoemulsion with different concentration of Tween 80 i.e., 5, 10, 15, 20 and 30%, (v/v)].

### 3.2 Antimicrobial activities of EuNE

#### 3.2.1 Antibacterial activity of EuNE

The results of antibacterial activities of the prepared EuNEs are summarized in Table 3, and shown in Fig. S1. The prepared EuNEs display their corresponding inhibition zones against three tested bacteria- *Escherichia coli, Bacillus subtilis*, and *Staphylococcus aureus*, and did not show inhibitory effects on *Bacillus subtilis*. However, EuNEs with higher concentrations of tween 80 (15–30%) exhibited significant antibacterial activity against *Staphylococcus aureus* and *Escherichia coli*. This significant bacterial inhibition were observed in nanoemulsions prepared with higher concentrations of Tween 80, which could be attributed to smaller size of the micelles, facilitating faster penetration of active compounds through the bacterial cell membrane (Hou et al., 2021). In retrospect, the highest zones of inhibition were observed for EuNE-30 against *Staphylococcus aureus* (35.99 mm) and *Escherichia coli* (32.99 mm), while average antibacterial activity was noted for EuNE-15 against *Staphylococcus aureus* (30.98 mm) and *Escherichia coli* (27.99 mm). Particularly, EuNE-30 demonstrated a significant zone of inhibition against both *Staphylococcus aureus* and *Escherichia coli*. The effectiveness of the inhibitors appears to increase with higher concentration of tween 80 irrespective of the bacterial species. It is well known that the surfactant and oil phase ratio play major role on the droplet size, and increase in surfactant (Tween 80) concentration led to the formation of smaller droplets in nanoemulsions. Similar results have also been reported where higher antibacterial activity of EuNE stabilized using Tween 80 was observed against *S. aureus, S. choleraesuis, E. coli, S. epidermis, S. mutans,* and *K. pneumoniae* (Fu et al., 2022).

**Table 3:** Antibacterial performance of the prepared EuNEs.

Nanoemulsion	Zone of inhibition (mm)		
	*Bacillus subtilis*	*Staphylococcus aureus*	*Escherichia coli*
EuNE-5 – (1)	–	–	–
EuNE-10 – (2)	–	–	–
EuNE-15 – (3)	–	30.98 ± 0.02^a^	27.99 ± 0.06^a^
EuNE-20 – (4)	–	32.59 ± 0.06^b^	30.98 ± 0.10^b^
EuNE-30 – (5)	–	35.99 ± 0.06^c^	32.99 ± 0.02^c^

The values are of triplicate assessment given as means ± SD, and the lower-case superscript letters indicate that they are significantly different (*p* < 0.05). [EuNE; Eugenol nanoemulsion with different concentration of Tween 80 i.e., 5, 10, 15, 20 and 30%, (*v*/v)].

#### 3.2.2 Antifungal activity of EuNE

**Fig. 1:**
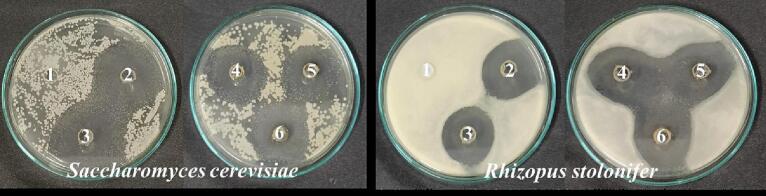
Antifungal assay of EuNEs against fungus (A) *S. cerevisiae*, and (B) *R. stolonifera* (in which 1, 2, 3, 4, 5, and 6 represent distilled water, EuNE-5, EuNE-10, EuNE-15, EuNE-20, EuNE-30, respectively) [EuNE; Eugenol nanoemulsion with different concentration of Tween 80 i.e., 5, 10, 15, 20 and 30%, (*v*/v)].

The results of antifungal activities of the EuNEs tested against two fungi i.e., *Saccharomyces cerevisiae, and Rhizopus stolonifer* are presented in Fig. 1 and Table S1, showing excellent antifungal performance of the prepared EuNEs against the tested fungi. Like the antibacterial performance, EuNE-30 also showed the highest inhibition compared to all other nanoemulsions studied in this work. The control blank sample did not show any inhibition zone as expected. The diameter of the zones of inhibition of EuNE-30 against *Saccharomyces cerevisiae* and *Rhizopus stolonifera* were 35.56 mm and 37.59 mm, respectively, while EuNE-5 induced zones of inhibition against the same fungi were 28.95 mm and 29.71 mm, respectively. A similar fungal inhibition pattern was observed by Basumatary, Kalita, et al., 2022, Basumatary, Mukherjee, et al., 2022 with isolated fungi (Basumatary, Mukherjee, et al., 2022) and Li, Zhao, Li, & Zhou, 2021 against *Penicillium Italicum* when exposed to eugenol nanoemulsions (Li et al., 2021).

### 3.3 Characterisation of the developed coating formulations

#### 3.3.1 pH, viscosity, and DPPH inhibition

Among the different nanoemulsions prepared, EuNE-20 was selected for the development of the coating formulations because of its stability and high antimicrobial properties. Table 4 summarises the pH, viscosity, and antioxidant activity of the developed shellac and carnauba wax-based coating formulations. The pH of all the developed coating solutions ranges between 8.08 and 8.43, which is slightly basic in nature, except for the AVG only (5.31 ± 0.1) which is slightly acidic in nature. No change in pH of the coating formulations was observed upon addition of EuNE and AVG in the wax matrix. The viscosity of all the coating formulations is higher than only AVG-2 coating formulation, however, the SW + CW had the highest viscosity. According to the obtained DPPH inhibition% results, the AVG-2 coating formulation showed the highest antioxidant capacity for scavenging DPPH radicals, with 88.14% of DPPH inhibition, whereas, SW + CW showed the lowest antioxidant capacity, with 12.82% of DPPH inhibition. Although the scavenging activity of the coating formulation varied significantly, they all exhibited reasonable antioxidant properties. Secondary metabolites, including flavonoids and other plant phenolics present in the plant extracts (such as essential oil) are mainly accountable for the antioxidant activities (da Silva Andrade et al., 2018).

**Table 4:** pH, viscosity, antioxidant activities of the developed coating formulations.

Coating formulations	pH value	Viscosity (cP)	DPPH inhibition (%)
SW + CW	8.43 ± 0.05^a^	20.0 ± 0.13^a^	12.82 ± 0.37^a^
SW + CW/EuNE-20	8.19 ± 0.18^ab^	10.0 ± 0.23^b^	24.61 ± 0.28^b^
SW + CW/AVG-2	8.39 ± 0.13^ab^	17.5 ± 0.26^c^	55.88 ± 0.54^c^
SW + CW/EuNE-20/AVG-2	8.08 ± 0.08^b^	15.0 ± 0.31^d^	83.26 ± 0.35^d^
AVG-2	5.31 ± 0.10^c^	5.0 ± 0.28^e^	88.14 ± 0.41^e^

The values are of triplicate assessment given as means ± SD, and the lower-case superscript letters indicate that they are significantly different (*p* < 0.05). (SW; Shellac wax, CW; Carnauba wax, EuNE-20; Eugenol nanoemulsion with 20% (*v*/v) Tween 80, and AVG; *Aloe vera* gel).

#### 3.3.2 Antibacterial activity of the developed coating formulations

**Fig. 2:**
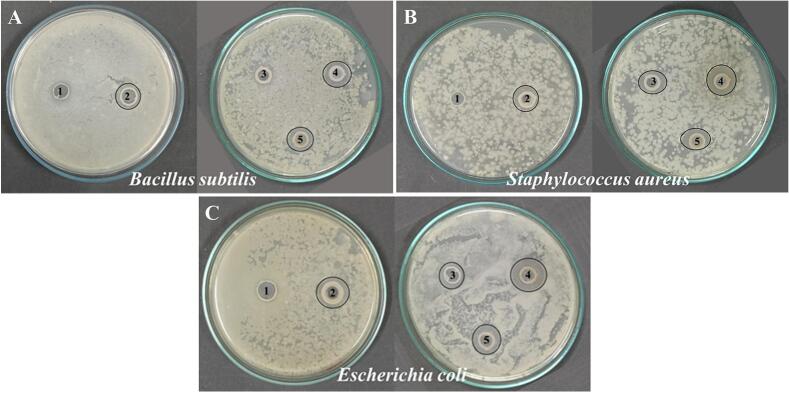
Antibacterial activity of the developed coating formulation against; (A) *B. subtilis,* (B) *E. aerogenes,* (C) *E. coli,* and (D) *S. aureus* (in which 1, 2, 3, 4, and 5 represent SW + CW, SW + CW/EuNE-20, SW + CW/AVG-2, SW + CW/EuNE-20/AVG-2, AVG-2, respectively) [SW; Shellac wax, CW; Carnauba wax, EuNE-20; Eugenol nanoemulsion with 20% (*v*/v) Tween 80, and AVG; *Aloe vera* gel].

The antibacterial activities of the coating formulations are represented as the size of the zone of inhibition (mm) as given in Table S2 and Fig. 2. Results indicate that only a few formulations exhibited antibacterial activity against the tested bacteria. Among them, SW + CW/EuNE-20/AVG-2 (4) displayed slightly stronger antibacterial activity than other coating formulations against all three tested bacteria, with inhibition zone sizes of 16.27 mm against *B. subtilis*, 18.55 mm against *S. aureus*, and 17.78 mm against *E. coli*. It is noteworthy that the addition of EuNEs and AVG into the coating formulations improved the inhibitory effects slightly. However, the coating formulation containing AVG, namely SW + CW/AVG-2 (3) showed no inhibitory effects against *B. subtilis* bacteria, while it exhibited inhibitory effects against other bacterial species such as *S. aureus* (14.78 mm) and *E. coli* (14.24 mm). The higher zones of inhibition observed could be attributed to the combination of EuNE and AVG, both of which have demonstrated significant antibacterial activity against different bacterial strains, as reported by several authors (Melendez-Rodriguez et al., 2019; Mirdehghan & Valero, 2017). On the other hand, the SW + CW (1) coating did not show inhibitory effects against the tested bacteria. This lack of effectiveness is attributed to the reduced activity of wax. One possible explanation provided is the low diffusion of wax compounds due to the diverse components of the coating's structure. This lower diffusion restricts the migration of oils from the solution, potentially leading to the ineffectiveness of the coating against the bacteria (Gutiérrez-Pacheco et al., 2020). It may be interesting to note that carnauba and shellac wax-based coating containing lemongrass oil also exhibited similar inhibition against *E. coli* O157:H7 growth (Jo et al., 2014).

### 3.4 Assessment of effects of coating application on the shelf-life of lemon fruit

#### 3.4.1 Weight loss

**Fig. 3:**
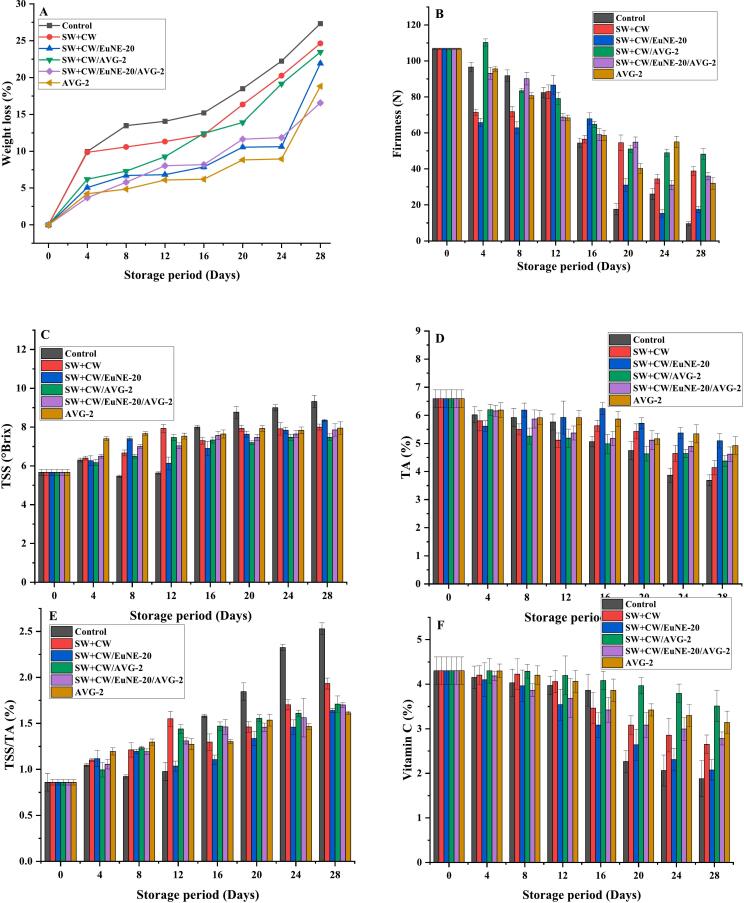
Effects of coating on (A) weight loss, (B) firmness, (C) TSS, (D) TA (E) TSS/TA, and (F) vitamin C contents of the lemon fruit during ambient storage for 28 days [SW; Shellac wax, CW; Carnauba wax, EuNE-20: Eugenol nanoemulsion with 20% (*v*/v) Tween 80, and AVG; *Aloe vera* gel].

The weight loss process occurs as water vapor diffuses across the skin due to the gradient in water pressure, and fruits generally lost weight quickly during storage. The percentage weight loss of coated and control lemon samples during storage is shown in Fig. 3(A). The weight loss of all the fruit samples gradually increased throughout the storage period, whereas the control sample had the higher weight loss compared to the coated ones. Among all coated lemon fruits, SW + CW/EuNE-20/AVG-2 coated fruits showed significantly lower weight loss from 27.33% to 16.56% (about 10.77%) after 28 days of storage. The coating offers a semi-permeable barrier on the fruit surface, which reduces weight loss in the coated fruits. Devi et al., 2023 reported that coating formulations incorporating carnauba wax emulsions and neem oil nanoemulsions applied to citrus fruits resulted in a 25% reduction in the rate of weight loss over a specific period of time (Susmita Devi, Kalita, Mukherjee, & Kumar, 2022). Álvarez-Barreto et al. (2023) observed similar results in strawberry fruits treated with carnauba wax-based edible coatings containing *aloe vera* gel where all coated fruits exhibited a weight loss percentage below 10% (Álvarez-Barreto et al., 2023). On the other hand, Khorram and Ramezanian (2021) reported that shellac coating enriched with cinnamon essential oil reduced weight loss by 52% and maintained the quality of ‘Thomson navel’ orange fruit for up to 21 days (Khorram & Ramezanian, 2021).

#### 3.4.2 Firmness

Firmness is associated with the overall fruit quality and freshness that influences consumer acceptance, as firmer fruits tend to be perceived as fresher, more juicy, crisp and crunchy (Thakur et al., 2019). Fig. 3(B) shows impacts of coating on firmness of the control and coated fruits. The firmness of all the fruit samples reduced during the storage duration. However, the coated fruits maintained a significantly higher firmness in comparison to the uncoated samples probably due to the lower moisture losses. The firmness in the coated lemon fruits were SW + CW (∼38 N), SW + CW/EuNE-20 (∼17 N), SW + CW/AVG-2 (∼48 N), SW + CW/EuNE-20/AVG-2 (∼36 N), and AVG-2 (∼ 32 N), whereas the uncoated control had about 10 N only after 28-days of storage. Incorporation AVG and EuNE in the wax coating formulations enhanced the firmness of the coated fruits, and among all the samples, SW + CW/AVG-2 coated fruits had the maximum firmness (∼48 N) after 28-days (reduced by ∼50% only). The increased firmness observed in the coated lemon suggests that the coating effectively suppressed enzymatic and metabolic activities, resulting in reduced water loss and tissue damage within the fruit due to decreased gas exchange and ethylene production facilitated by the coating material (Thakur et al., 2019). Yan et al., reported that while fruit firmness typically decreased continuously during storage; grapefruit coated with shellac wax significantly slowed down the fruit softening, and after 8 weeks of storage, coated fruit exhibited 8–13% greater firmness compared to the control (Yan, Zhang, Hu, Deng, & Ritenour, 2020). Other researchers have also reported similar findings on higher retention of fruit firmness by using shellac/carnauba wax based coatings on various fruits such as mango (Ma et al., 2021), papaya (Miranda et al., 2022) and litchi (Nanglia et al., 2022), etc.

#### 3.4.3 Total soluble solids (TSS) content

TSS primarily consist of sugars and soluble minerals that are found in fruit juice, which have major influence on the flavour of the fruits. The TSS content of coated lemons exhibited a consistent rise in the TSS levels throughout the storage period that indicate sweetness retention in case of coated fruits (Fig. 3(C)). However, a gradual decline in TSS of the control samples was observed during day 8 to day 12 of storage, and a sudden increase in TSS was observed after day12. Maximum TSS was observed in control samples (9.32°Brix), followed by SW + CW/EuNE-20 (8.34°Brix), SW + CW (8°Brix), AVG-2 (7.95°Brix), SW + CW/EuNE-20/AVG-2 (7.85°Brix), and SW + CW/AVG-2 (7.47°Brix) at day 28. Coatings, particularly AVG-2, SW + CW/EuNE-20/AVG-2, and SW + CW/AVG-2 were more effective in maintaining TSS content similar to report of Nie, Huang, Chen, Wan, & Chen, 2020 (Nie et al., 2020). Similarly, apples coated with carnauba wax and 1-methylcyclopropene retained significantly higher TSS content compared to control during storage (Chen, Jiang, Zeng, Huo, & Li, 2020).

#### 3.4.4 Titratable acidity (TA)

Citric and malic acids are the primary organic acids present in lemon fruits, contributing to their acidity, flavour and taste. The amount of acid in the fruits decreases over time, likely because of the oxidation of organic acids during storage, a process that naturally occur as fruit ripens (Cofelice, Lopez, & Cuomo, 2019). Fruit coatings can efficiently reduce respiration and oxidation leading to slower conversion of organic acids into sugars. The TA values of all the lemon fruits significantly decreased throughout the storage period, however the coated fruit samples showed a slower reduction compared to the uncoated fruit samples (Fig. 3(D)). In case of control sample TA declined rapidly from 6.59% to 3.68% during storage, signifying faster senescence and ripening. In the coated fruits, however, the TA was maintained up to 28-days of storage. The fruit samples coated with SW + CW//EuNE-20 and AVG-2 had highest TA value of 5.09% and 4.9%, respectively that was maintained even at day 28 of the storage. This could be due to the high barrier properties of the wax-based coating, and high antioxidant ability of AVG. Babarabie, Sardoei, Jamali, and Hatami (2024) observed that orange fruits treated with the highest concentration of carnauba wax coating exhibited the highest fruit acidity levels (∼4.5%), followed by those treated with comparatively lower concentration of carnauba wax (around 2.5–3%), compared to the control during storage (Babarabie et al., 2024). Similar results were reported by the other researchers while studying wax-based composite coatings on strawberries (Oliveira Filho, 2022), and ‘Fuji’ apple (Chen et al., 2020), etc. In another study, Oliveira Filho et al. (2023) observed a decrease in TA values of papaya fruit coated with carnauba wax nanoemulsion combined with various essential oils, attributed to the utilization of organic acids such as citric acid during respiration (J. G. Oliveira Filho et al., 2023).

#### 3.4.5 Maturity index (TSS/TA)

The maturity of all the lemon fruits increased with the time of storage, representing their ripening (Fig. 3(E)). Maturity gain was however slower in the coated fruits. The lowest TSS/TA value was observed in samples coated with SWCW//EuNE-10 (1.63%) and AVG-2 (1.61%) after day 28 of storage, primarily due to rise in TA values. Incorporation of EuNE and AG in the shellac-carnauba wax coating positively influenced, delaying maturity and ripening rate thus resulting in shelf-life extension of the coated fruits. In an earlier work we reported that fruits coated with wax-based coatings maintained a better maturity index compared to uncoated fruits throughout the storage period. The coated fruits exhibited a lower TSS/TA ratio (22.51) compared to the control samples (24.8) at the end of the 28-day storage period, indicating that the coatings improved the postharvest-life of the fruits by delaying maturity and reducing the ripening rate (Devi et al., 2023). The results of other researchers showed similar behaviour in case of wax-based coatings on oranges (Khorram, Ramezanian, & Hosseini, 2017a; Khorram, Ramezanian, & Hosseini, 2017b), and ‘Kinnow’ mandarins (Khorram et al., 2017a, Khorram et al., 2017b).

#### 3.4.6 Ascorbic acids (Vitamin C) content

Ascorbic acids are widely recognized as one of the essential water-soluble antioxidants. Coating on fruits form a gas barrier on their surface that decreases autoxidation of ascorbic acid in the presence of oxygen. The ascorbic acid level of all coated and uncoated lemons decreased during storage; however, the application of coatings effectively retarded the decrease compared to uncoated fruits, particularly in lemon fruits coated with SW + CW/AVG-2 (3.51%) and AVG-2 (3.1%) giving the highest ascorbic acid content after 28 days of storage (Fig. 3(F)). This finding is in accordance with the results reported by Chen et al. (2020), who observed that ‘Fuji’ apples coated with carnauba wax and 1-methylcyclopropene retained higher ascorbic acid content compared to control fruit during 105 days of storage. The author suggested that the higher ascorbic acid observed in the study may be attributed to the synergistic effect of 1-methylcyclopropene and carnauba wax nanoemulsion in delaying the ripening of the fruits (Chen et al., 2020). Similarly, Babarabie et al. (2024) conducted a study where the highest concentration of vitamin C (∼1–1.2 mg/g) was found in orange fruits coated with carnauba wax during storage (Babarabie et al., 2024). Similar studies have demonstrated the efficacy of wax-based coatings for improving the ascorbic acid content of coated strawberries during storage (Oliveira Filho, 2022).

#### 3.4.7 Effects of coating on visual appearance of the coated fruits

**Fig. 4:**
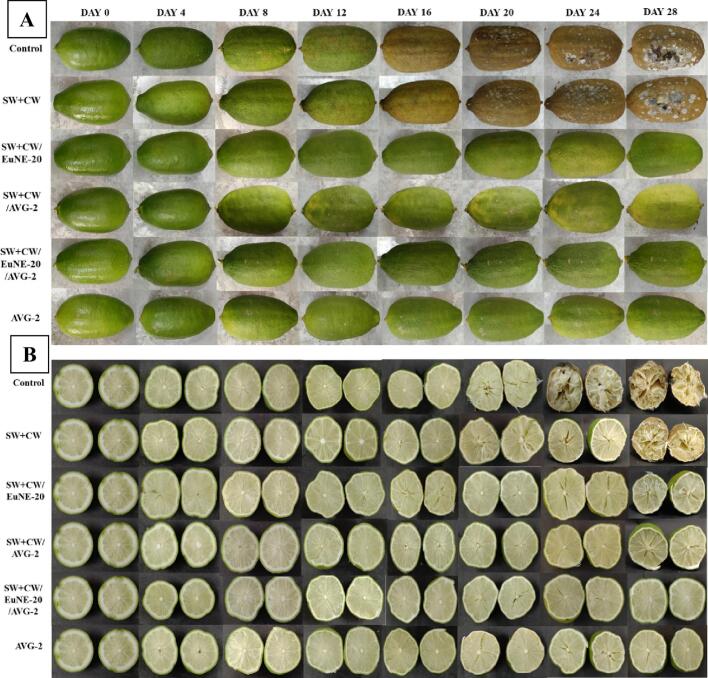
Visual appearance of the (A) whole coated lemon, and (B) internal tissue of the cut-lemon during 28 days at ambient storage [SW; Shellac wax, CW; Carnauba wax, EuNE-20: Eugenol nanoemulsion with 20% (v/v) Tween 80, and AVG; *Aloe vera* gel].

The coated fruits were visually analysed by assessing their change in skin color, growth of fungus and lesions on fruit surface, ripening and deterioration, and ultimately the overall quality of the lemon during storage for 28 days (Basumatary et al., 2021). The skin color of the coated and uncoated lemons changes from green to yellowish to brown during storage (Fig. 4). After 12 days of storage, uncoated and SW + CW coated lemon significantly changed their color and turn rapidly brown, and by the day 20, the samples began to show signs of mould growth. However, lemon coated with SW + CW/EuNE-20, SW + CW/AVG-2, SW + CW/EuNE-20/AVG-2, and AVG-2 maintained the fresh appearance and prevented from mould spoilage even after storage of 20 days, as shown in Fig. 4A. The internal tissues of the coated fruits also showed similar results as can be in observed from the cut-fruits shown in Fig. 4B. Figure show that the damage affects 50%–90% of the internal tissues in the control and SW + CW coated fruits, but no sign of internal tissue damage was observed in case of SW + CW/EuNE-20, SW + CW/AVG-2, SW + CW/EuNE-20/AVG-2, AVG-2 coated lemon even after 28 days of storage. The nanocomposite coatings' defensive properties may be the reason for this, since they contain antibacterial and antioxidant ingredients (EuNE and *aloe vera* gel). Visual appearance of lemon fruits indicated that the developed coatings can control fruit color changes, texture, internal tissue damage, and can extend shelf life of the lemon fruits by at least 20 days, whereas uncoated and SW + CW coated fruits showed signs of decay after 12 days of storage. These results are consistent with the other reports on the application of wax-based edible coatings containing natural active compounds (Aguirre-Joya et al., 2019).

#### 3.4.8 Sensory analysis

The key attributes of the fruits that matter most to consumers are their appearance, flavour, and aroma, as these factors directly contribute to the overall acceptance of the fruits. The results of sensory evaluation are presented in Table 5 showing that the application of coating did not adversely affect the quality attributes of the lemon (taste, aroma, appearance, and overall acceptability). During the sensory test, the lemon sample treated with SW + CW/EuNE-20/AVG-2 received the best scores compared to the other samples. The control sample started to exhibit a decline in its sensory properties after day-16 of storage, whereas, the coated fruits maintained their acceptability throughout the 28 days of storage period, except for the SW + CW coated sample, which significantly deteriorate after day 12 of storage. The visual appearance played a crucial role in evaluating the attractiveness of the fruit samples, serving as a significant criterion in the sensory evaluation process. At the end of the 28 days storage period, the lemon samples coated with SW + CW/EuNE-20/AVG-2, received the highest appearance scores (score 6.0), followed by AVG-2 (score 5.8), SW + CWEuNE-20 (score 5.4), and SW + CW/AVG-2 (score 5.2). The aroma scores for both the control lemon and SW + CW coated lemons fell below the acceptable limit (score 5.0) within 12 days of storage. In contrast, the other coated lemons were acceptable till 28-days of ambient storage. The overall acceptability score of the uncoated samples received a score of 3.5 after day 16, which falls below the acceptable limit. However, the coated samples were deemed acceptable and received higher scores (score > 6.0), except for the SW + CW coated lemon, which received the lowest score of 4.5 among the treated samples. This suggests that the developed composite coating formulations effectively preserved the sensory attributes of lemon for a period of at least 20 days in storage. Also, the incorporation of EuNEs and AG into the composite coating notably enhanced the sensory properties of the lemon fruits by reducing microbial spoilage and preserving the sensory properties of the coated lemon fruits.

**Table 5:** Sensory analysis of shellac and carnauba wax coated lemon.

Parameter	Day	Control	SW + CW	SW + CW/EuNE-20	SW + CW/AVG-2	SW + CW/EuNE-20/AVG-2	AVG-2
Taste	0	8.7 ± 0.03^a^	8.5 ± 0.02^a^	8.7 ± 0.03_a_	8.8 ± 0.02^a^	8.0 ± 0.05^a^	8.6 ± 0.05^a^
	4	8.0 ± 0.07^a^	8.0 ± 0.07^a^	8.3 ± 0.03_a_	8.6 ± 0.05^a^	8.7 ± 0.03^a^	8.5 ± 0.02^a^
	8	7.0 ± 0.06^a^	8.0 ± 0.07^a^	8.0 ± 0.07^a^	8.0 ± 0.07^a^	8.6 ± 0.05^ab^	8.1 ± 0.57^a^
	12	6.0 ± 0.04^b^	7.0 ± 0.06^ab^	7.9 ± 0.05^ab^	7.2 ± 0.07^ab^	8.6 ± 0.05^ab^	8.0 ± 0.07^ab^
	16	3.2 ± 0.03^b^	4.4 ± 0.02^b^	7.3 ± 0.02^a^	7.0 ± 0.03^a^	8.4 ± 0.07^a^	7.6 ± 0.03^a^
	20	2.0 ± 0.08^b^	3.2 ± 0.03^b^	6.7 ± 0.03^a^	6.7 ± 0.3^a^	8.0 ± 0.07^a^	7.0 ± 0.03^a^
	24	0	1.0 ± 0.05^b^	6.2 ± 0.04^a^	6.1 ± 0.04^a^	7.6 ± 0.03^a^	6.2 ± 0.04^a^
	28	0	0	6.0 ± 0.04^a^	6.0 ± 0.04^a^	6.8 ± 0.03^a^	6.0 ± 0.04^a^
Visual appearance	0	8.4 ± 0.08^ab^	8.5 ± 0.05^ab^	8.5 ± 0.50^ab^	8.7 ± 0.05^ab^	8.7 ± 0.08^ab^	8.4 ± 0.05^b^
	4	7.5 ± 0.05^d^	7.8 ± 0.05^c^	8.1 ± 0.50^b^	8.4 ± 0.05^a^	8.6 ± 0.03^a^	8.2 ± 0.03^ab^
	8	6.8 ± 0.05^d^	7.3 ± 0.03^c^	7.7 ± 0.30^b^	7.8 ± 0.05^b^	8.5 ± 0.03^a^	7.8 ± 0.03^b^
	12	5.7 ± 0.03e	6.8 ± 0.05d	7.6 ± 0.50^b^	7.2 ± 0.05^c^	8.4 ± 0.03^a^	7.6 ± 0.05^b^
	16	2.8 ± 0.05^d^	3.6 ± 0.05^c^	6.7 ± 0.30^b^	6.5 ± 0.03^b^	7.8 ± 0.05^a^	6.8 ± 0.05^b^
	20	1.8 ± 0.03^e^	2.7 ± 0.05^d^	6.4 ± 0.30^c^	6.3 ± 0.03^c^	7. 3 ± 0.05^a^	6.7 ± 0.03^b^
	24	0	1.4 ± 0.05^d^	5.8 ± 0.50^c^	5.8 ± 0.05^c^	6.8 ± 0.05^a^	6.4 ± 0.05^b^
	28	0	0	5.4 ± 0.10^c^	5.2 ± 0.05^c^	6.0 ± 0.05^a^	5.8 ± 0.03^b^
Aroma	0	8.6 ± 0.05^ab^	8.4 ± 0.05^bc^	8.3 ± 0.05^c^	8.7 ± 0.05^a^	8.8 ± 0.05^a^	8.7 ± 0.05^a^
	4	7.6 ± 0.05^c^	7.5 ± 0.28^c^	8.1 ± 0.03^bc^	8.5 ± 0.03^ab^	8.5 ± 0.1^ab^	8.5 ± 0.05^ab^
	8	6.0 ± 0.05^c^	6.6 ± 0.08^b^	7.5 ± 0.18^a^	7.2 ± 0.03^a^	7.7 ± 0.12^a^	7.6 ± 0.08^a^
	12	6.0 ± 0.05^c^	6.6 ± 0.08^b^	7.5 ± 0.18^a^	7.2 ± 0.03^a^	7.7 ± 0.12^a^	7.6 ± 0.08^a^
	16	4.1 ± 0.06^c^	4.3 ± 0.03^c^	6.8 ± 0.05^b^	6.8 ± 0.05^a^	7.4 ± 0.05^a^	7.3 ± 0.05^a^
	20	2.1 ± 0.05^c^	3.0 ± 0.05^b^	6.4 ± 0.26^a^	6.2 ± 0.10^b^	6.8 ± 0.03^a^	7.0 ± 0.10^a^
	24	1.6 ± 0.03^e^	1.6 ± 0.08^d^	5.7 ± 0.05^b^	5.3 ± 0.03^c^	6.4 ± 0.08^a^	6.6 ± 0.08^a^
	28	0	1.2 ± 0.05^d^	5.5 ± 0.08^b^	5.1 ± 0.03^c^	6.1 ± 0.06^a^	6.5 ± 0.03^a^
Overall acceptability	0	8.4 ± 0.03^ab^	8.6 ± 0.03^ab^	8.7 ± 0.12^a^	8.4 ± 0.05^b^	8.4 ± 0.08^ab^	8.6 ± 0.03^ab^
	4	7.4 ± 0.05^b^	7.5 ± 0.06^b^	8.5 ± 0.05^a^	8.3 ± 0.05^a^	8.4 ± 0.08^a^	8.2 ± 0.05^a^
	8	6.6 ± 0.05^c^	6.6 ± 0.08^c^	7.7 ± 0.12^b^	7.7 ± 0.05^a^	8.3 ± 0.05^a^	7.8 ± 0.05^b^
	12	5.1 ± 0.05^c^	5.5 ± 0.05^d^	7.6 ± 0.05^ab^	7.3 ± 0.12^a^	7.8 ± 0.03^a^	7.4 ± 0.05^b^
	16	3.5 ± 0.05^d^	4.5 ± 0.05^d^	6.7 ± 0.12^c^	7.0 ± 0.08^ab^	7.5 ± 0.08^ab^	7.3 ± 0.05^b^
	20	1.3 ± 0.08^d^	2.8 ± 0.05^c^	5.8 ± 0.03^b^	6.7 ± 0.03^a^	6.9 ± 0.08^a^	6.6 ± 0.08^a^
	24	0	2.0 ± 0.10^c^	5.3 ± 0.03^b^	5.4 ± 0.08^b^	6.3 ± 0.08^a^	6.3 ± 0.12^a^
	28	0	1.1 ± 0.08^d^	4.9 ± 0.08^b^	4.3 ± 0.11^c^	5.6 ± 0.11^a^	5.4 ± 0.03^a^

The values are of triplicate assessment given as means ± SD, and the lower-case superscript letters indicate that they are significantly different (*p* < 0.05) within treatment, between different durations (days) of storage [SW; Shellac wax, CW; Carnauba wax, EuNE-20: Eugenol nanoemulsion with 20% (*v*/v) Tween 80, and AVG; *Aloe vera* gel].

## 4 Conclusions

In this study, efficacy of lipid-based sustainable coating formulations in maintaining postharvest quality and shelf-life extension of lemon fruits were assessed. The prepared oil-in-water (O/W) eugenol nanoemulsions (EuNEs) showed good antioxidant activities, and thus incorporated in wax matrix along with *Aloe vera* gel as active functional agents. The outcomes of the study showed that this developed coating met most of the specified requirements of edible coatings, which reduces fruit weight loss, maintains the tissue firmness, TSS, TA, and vitamin C content. The coating formulations, SW + CW/EuNE-20, SW + CW/EuNE-20/AVG-2, and only AVG-2, showed the most effective results in preventing fruit decay and prolonging the shelf life of lemon fruits for at least 20 days during storage. The incorporation of EuNEs and AVG not only reduced deterioration of lemon fruit during storage but also preserved the sensory qualities of the fruit within the acceptable limits. These developed coatings may replace existing synthetic wax-based coatings used for delaying deterioration of fruits and vegetables. Additional research is however necessary to explore the antimicrobial properties and toxicity of this developed natural wax-based coating formulation prior to its wide-spread application.

## CRediT authorship contribution statement

**Bhaswati Das:** Writing – original draft, Software, Investigation, Formal analysis. **L. Susmita Devi:** Writing – original draft, Methodology, Formal analysis, Data curation. **Joydeep Dutta:** Visualization, Resources, Conceptualization, Writing – review & editing. **Santosh Kumar:** Conceptualization, Funding acquisition, Resources, Supervision, Visualization, Writing – review & editing.

## Declaration of competing interest

Santosh Kumar reports financial support was provided by India Ministry of Science & Technology Department of Biotechnology. If there are other authors, they declare that they have no known competing financial interests or personal relationships that could have appeared to influence the work reported in this paper.

## Data Availability

Data will be made available on request.

## Appendix A Supplementary data

Supplementary material 1: Figure S1 Image 1

Supplementary material 2: Table S1 Image 2

Supplementary material 3: Table S2 Image 3
