# Supplementary material for: Eugenol and Aloe vera blended natural wax-based coating for preserving postharvest quality of Kaji lemon (Citrus jambhiri)
Source: Food Chem X. 2024 Apr 6;22:101349. doi: 10.1016/j.fochx.2024.101349 (PMC11016979; doi:10.1016/j.fochx.2024.101349)
Supplement: Supplementary file 1 — Supplementary material 1: Figure S1 [file mmc1.docx]

**Eugenol and *Aloe vera* blended natural wax-based coating for preserving postharvest quality of Kaji lemon (*Citrus jambhiri*)**

**Bhaswati Das^1^, L. Susmita Devi^1^, Joydeep Dutta^2,*^, and Santosh Kumar^1,*^**

^1^Department of Food Engineering and Technology, Central Institute of Technology Kokrajhar, Kokrajhar, Assam-783370, India

^2^Functional NanoMaterials Group, Department of Applied Physics, School of Engineering Sciences, KTH Royal Institute of Technology, Hannes Alfvéns väg 12, 114 19 Stockholm, Sweden

***Corresponding authors:**

Dr. Santosh Kumar**;** [s.kumar@cit.ac.in](mailto:s.kumar@cit.ac.in); ORCID: <https://orcid.org/0000-0003-3017-4872>

Prof. Joydeep Dutta; [joydeep@kth.se](mailto:joydeep@kth.se); ORCID: https://orcid.org/0000-0002-0074-3504


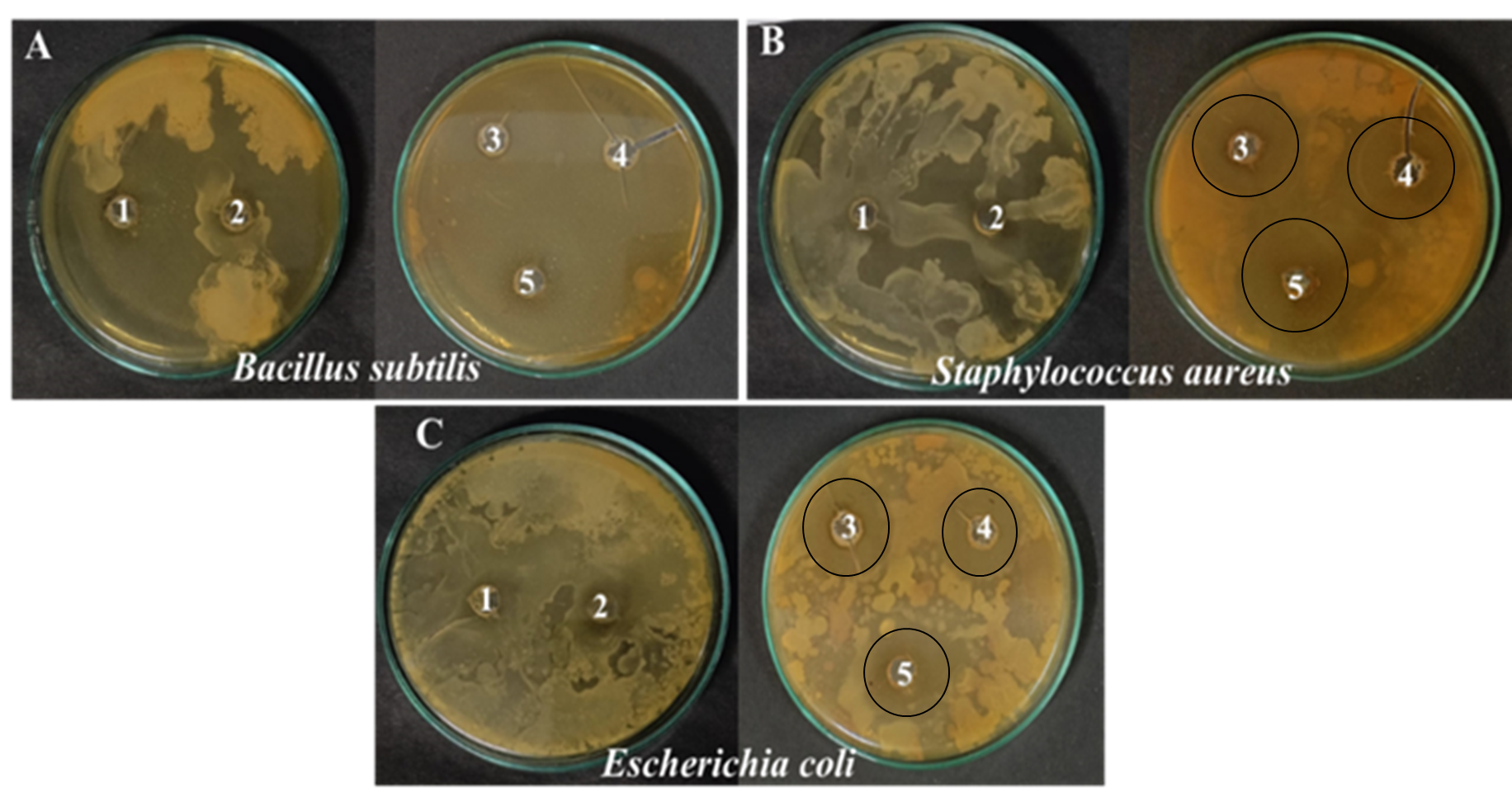


**Figure S1.** Antibacterial activity of EuNEs; (A) *Bacillus subtilis*, (B) *Staphylococcus aureus,* (C) *Escherichia coli* **[**EuNE: Eugenol nanoemulsion with different concentrations of Tween 80 i.e., 5, 10, 15, 20 and 30 %, (v/v)].
