# Supplementary material for: Eugenol and Aloe vera blended natural wax-based coating for preserving postharvest quality of Kaji lemon (Citrus jambhiri)
Source: Food Chem X. 2024 Apr 6;22:101349. doi: 10.1016/j.fochx.2024.101349 (PMC11016979; doi:10.1016/j.fochx.2024.101349)
Supplement: Supplementary file 2 — Supplementary material 2: Table S1 [file mmc2.docx]

**Eugenol and *Aloe vera* blended natural wax-based coating for preserving postharvest quality of Kaji lemon (*Citrus jambhiri*)**

**Bhaswati Das^1^, L. Susmita Devi^1^, Joydeep Dutta^2,*^, and Santosh Kumar^1,*^**

^1^Department of Food Engineering and Technology, Central Institute of Technology Kokrajhar, Kokrajhar, Assam-783370, India

^2^Functional NanoMaterials Group, Department of Applied Physics, School of Engineering Sciences, KTH Royal Institute of Technology, Hannes Alfvéns väg 12, 114 19 Stockholm, Sweden

***Corresponding authors:**

Dr. Santosh Kumar**;** [s.kumar@cit.ac.in](mailto:s.kumar@cit.ac.in); ORCID: <https://orcid.org/0000-0003-3017-4872>

Prof. Joydeep Dutta; [joydeep@kth.se](mailto:joydeep@kth.se); ORCID: https://orcid.org/0000-0002-0074-3504

**Table S1.** Antifungal analysis of the prepared EuNEs

| **Nanoemulsion** | **Zone of inhibition (mm)** | |
| --- | --- | --- |
|  | ***Streptococcus cerevisiae*** | ***Rhizopus stolonifer*** |
| 1 (Blank) | - | - |
| 2 (EuNE-5) | 28.95 ± 0.053^a^ | 29.71 ± 0.063^a^ |
| 3 (EuNE-10) | 29.97 ± 0.043^b^ | 30.48 ± 0.087^b^ |
| 4 (EuNE-15) | 33.52 ± 0.075^c^ | 32.25 ± 0.053^c^ |
| 5 (EuNE-20) | 35.05 ± 0.066^d^ | 36.32 ± 0.064^d^ |
| 6 (EuNE-30) | 35.56 ± 0.065^e^ | 37.59 ± 0.065^e^ |

The values are of triplicate assessment given as means ± SD, and the lower-case superscript letters indicate that they are significantly different (p < 0.05) [EuNE; Eugenol nanoemulsion with different concentrations of Tween 80 i.e., 5, 10, 15, 20 and 30 %, (v/v)].
