# Supplementary material for: Eugenol and Aloe vera blended natural wax-based coating for preserving postharvest quality of Kaji lemon (Citrus jambhiri)
Source: Food Chem X. 2024 Apr 6;22:101349. doi: 10.1016/j.fochx.2024.101349 (PMC11016979; doi:10.1016/j.fochx.2024.101349)
Supplement: Supplementary file 3 — Supplementary material 3: Table S2 [file mmc3.docx]

**Eugenol and *Aloe vera* blended natural wax-based coating for preserving postharvest quality of Kaji lemon (*Citrus jambhiri*)**

**Bhaswati Das^1^, L. Susmita Devi^1^, Joydeep Dutta^2,*^, and Santosh Kumar^1,*^**

^1^Department of Food Engineering and Technology, Central Institute of Technology Kokrajhar, Kokrajhar, Assam-783370, India

^2^Functional NanoMaterials Group, Department of Applied Physics, School of Engineering Sciences, KTH Royal Institute of Technology, Hannes Alfvéns väg 12, 114 19 Stockholm, Sweden

***Corresponding authors:**

Dr. Santosh Kumar**;** [s.kumar@cit.ac.in](mailto:s.kumar@cit.ac.in); ORCID: <https://orcid.org/0000-0003-3017-4872>

Prof. Joydeep Dutta; [joydeep@kth.se](mailto:joydeep@kth.se); ORCID: https://orcid.org/0000-0002-0074-3504

**Table S2.** Antibacterial analysis of the developed coating formulations

| **Coating solution** | **Zone of inhibition (mm)** | | |
| --- | --- | --- | --- |
|  | ***Bacillus subtilis*** | ***Staphylococcus aureus*** | ***Escherichia coli*** |
| 1 (SW+CW) | - | - | - |
| 2 (SW+CW/EuNE-20) | 13.46 ± 0.04^a^ | 11.14 ± 0.04^a^ | 14.47± 0.02^a^ |
| 3 (SW+CW/AVG-2) | - | 14.78 ± 0.03^b^ | 14.24 ± 0.02^b^ |
| 4 (SW+CW/EuNE-20/AVG-2) | 16.27 ± 0.06^b^ | 18.55 ± 0.02^c^ | 17.78 ± 0.10^c^ |
| 5 (AVG-2) | 15.47± 0.02^c^ | 17.27± 0.02^d^ | 15.24 ± 0.06^d^ |

The values are of triplicate assessment given as means ± SD, and the lower-case superscript letters indicate that they are significantly different (p < 0.05). [SW; Shellac wax, CW; Carnauba wax, EuNE-20; Eugenol nanoemulsion with 20 % (v/v) tween 80, and AVG; *Aloe vera* gel].
